# The secrets of the stability of the HIV-1 capsid

**DOI:** 10.7554/eLife.38895

**Published:** 2018-07-31

**Authors:** Martin Obr, Hans-Georg Kräusslich

**Affiliations:** Center of Infectious DiseasesHeidelberg UniversityHeidelbergGermany

**Keywords:** HIV-1, capsid, IP6, R18 pore, PF74, CA lattice, Human, Virus

## Abstract

Structural and biophysical studies help to follow the disassembly of the HIV-1 capsid in vitro, and reveal the role of a small molecule called IP6 in regulating capsid stability.

**Related research article** Mallery DL, Márquez CL, McEwan WA, Dickson CF, Jacques DA, Anandapadamanaban M, Bichel K, Towers GJ, Saiardi A, Böcking T, James LC. 2018. IP6 is an HIV pocket factor that prevents capsid collapse and promotes DNA synthesis. *eLife* **7**:e35335. doi: 10.7554/eLife.35335

**Related research article** Márquez CL, Lau D, Walsh J, Shah V, McGuinness C, Wong A, Aggarwal A, Parker MW, Jacques DA, Turville S, Böcking T. 2018. Kinetics of HIV-1 capsid uncoating revealed by single-molecule analysis. *eLife* **7**:e34772. doi: 10.7554/eLife.34772

When HIV-1 enters a cell during infection, its genetic information is encased in a protein shell, termed the capsid. Once in the cell, the HIV-1 RNA genome gets copied into cDNA, in a process known as reverse transcription. This cDNA then enters the nucleus, where it can be integrated into the DNA of the cell. While reverse transcription can start within the protected environment of the capsid, the integration of cDNA requires the protein shell to disassemble or ‘uncoat’. Ensuring that the capsid disassembles at the right time and place is crucial for the virus (Forshey et al., 2002), but little is known about how this process may be regulated. Different models exist, suggesting gradual uncoating – either en route to the nucleus, or directly at the nuclear membrane (Campbell and Hope, 2015) – but there is much that we do not understand. Moreover, what happens may also depend on the host cell.

The mature HIV-1 capsid is a cone-shaped structure made of 1,000–1,500 copies of a single protein, known as CA (Figure 1A). They are organized into 200–250 CA hexamers, and 12 pentamers (Figure 1B; Pornillos et al., 2011; Mattei et al., 2016). The center of each hexamer contains a pore, which is lined by six positively charged arginine 18 residues (R18; Figure 1C). This R18 pore is covered by a motif known as β-hairpin that can adopt different conformations, thereby opening and closing the pore. It has been suggested that this pore allows deoxyribonucleotides (dNTPs), which are needed for reverse transcription, to enter the capsid (Jacques et al., 2016). Now, in two papers in eLife, researchers in Australia and the UK report new insights into the uncoating process and the role of the R18 pore.

**Figure 1.**
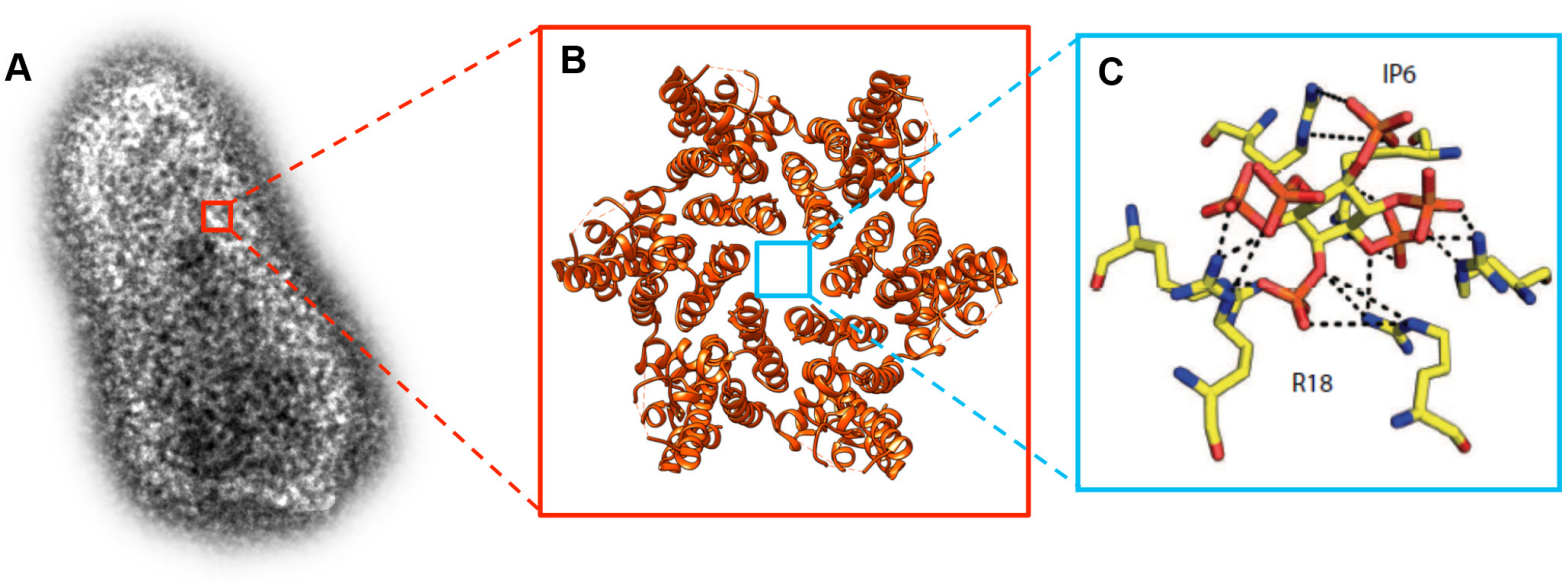
Molecular structure of the HIV-1 capsid and the small molecule IP6 binding to the R18 pore. (**A**) Transmission electron microscopy image of an authentic negative stained HIV-1 capsid. The capsid is formed of hundreds of CA proteins organized in hexamers and pentamers. (**B**). The structure of a CA hexamer, revealed by X-ray crystallography (PDB ID: 3H47). (**C**). Detail of the small molecule IP6 (IP6; in the center) binding to a ring of six arginines (R18; on the outside) within the R18 pore at the center of a CA hexamer. IP6 contains six negatively charged phosphate groups , which are coordinated by the positively charged guanidino groups of the R18 side chains. Dashed lines indicate interactions between the guanidino and phosphate groups (Panel C is adapted from Mallery et al., 2018).

In the first paper, Leo James of the MRC Laboratory of Molecular Biology, Till Böcking of UNSW Sydney and co-workers – including Donna Mallery as first author and researchers from University College London – searched for small molecules that can bind to the R18 pore (Mallery et al., 2018). They found that ATP attached at the same position as dNTPs, and with similar efficiency. Searching for other ligands, Mallery et al. found that a molecule called IP6 (short for inositol hexakisphosphate) binds in the center of the pore, stabilizing the CA hexamer (Figure 1C). Surprisingly, despite blocking a pore that can conduct dNTPs, ATP and IP6 stimulated reverse transcription inside the capsid rather than inhibiting it.

Both ATP and IP6 were detected in purified HIV-1 particles, and more IP6 was found on HIV-1 than expected from levels within the cell, suggesting that the virus recruits this molecule. Quantitative analysis revealed that each virus carries about 300 IP6 molecules, with most of them being on the capsid itself. This number is remarkably similar to the number of CA hexamers per mature capsid, indicating that each R18 pore may carry an IP6 molecule. It is possible that IP6 could stabilize the incoming HIV-1 capsid on its way to the nucleus, since it can bind and stabilize the CA hexamers. However, this needed to be proven by looking at the effect of IP6 on capsid integrity and disassembly.

In the second paper, Böcking and colleagues at UNSW Sydney and the University of Melbourne – including Chantal Márquez as first author (Márquez et al., 2018) – report that they have developed two ways to monitor how a HIV-1 capsid uncoats in vitro: one detects the moment when the capsid starts to lose its integrity, while the other observes the gradual loss of CA proteins as the capsid degrades over time.

When the lipid envelope that protects the viral capsid outside of the cell was taken away, and no further IP6 was added, the experiments showed that a large number of capsids were defective, opening immediately and decaying catastrophically. The intact capsids displayed a half-life of 7–10 minutes after removal of the membrane, with the CA lattice being lost over an additional 1–2 minutes. These results are consistent with models suggesting that, unless other molecules stabilize them, capsids quickly disintegrate as soon as some of their building blocks are removed.

The groups of Böcking and James then joined forces to determine the integrity of the capsid when additional IP6 was given as the lipid envelope was removed. These experiments revealed a remarkable increase in the capsid half-life, from 7–10 minutes to 10 hours, upon IP6 binding. An intriguing result was obtained using a small molecule called PF74, which is an antiviral drug that binds to CA hexamers but distant from the R18 pore (Price et al., 2014). In vitro, PF74 stabilizes assembled capsid-like structures (Bhattacharya et al., 2014), while apparently destabilizing capsids in infected cells (Shi et al., 2011). This discrepancy can now be reconciled: PF74 caused capsids to quickly lose their integrity, even in the presence of stabilizing IP6 molecules. However, it stabilized the remaining CA lattice. These findings may explain how PF74 can impair reverse transcription in vitro and in cell culture.

The results indicate that IP6 and similar molecules can keep the HIV-1 capsid stable, and may thus be essential for the virus to successfully replicate. Recruitment into the virus underscores the importance of IP6, while ATP may play a role as well, given its high levels in the cell. The R18 pore that binds IP6 is only present when the capsid is mature, but this maturation process takes place once the virus has left the cell. Yet, it is possible that IP6 is already recruited during formation of the virus particle, as in vitro experiments show that the molecule promotes assembly of the immature lattice (Campbell et al., 2001). After being recruited during assembly of the immature particle, IP6 would then serve a second function in newly infected cells.

This work considerably furthers our understanding of how HIV-1 uncoats, but it also raises important questions. For instance, can R18 pores both conduct dNTPs and bind ligands that stabilize capsids? Since reverse transcription is most strongly enhanced when the levels of ATP or IP6 are high, R18 pores may not be essential to import dNTPs, unless specialized subsets of pores exist.

It also remains unclear what causes the R18 pore to lose IP6, and whether this event starts the disassembly of the capsid. One hypothesis is that a conformational change of the β-hairpin may eject the small molecule and initiate uncoating, but the trigger is unknown.

Finally, it may be possible for ‘natural’ ligands to fulfill the stabilizing role of PF74. Certain proteins found in cells can attach to sites that overlap with the one occupied by PF74 (Jacques et al., 2016; Bhattacharya et al., 2014; Price et al., 2014). In addition, several groups have suggested the existence of partially disassembled CA lattices (Campbell and Hope, 2015), which may be stabilized by capsid-binding proteins similar to PF74. Why this would be advantageous for the virus is another puzzling question.
